# Enterovirus D-68 Infection of Primary Rat Cortical Neurons: Entry, Replication, and Functional Consequences

**DOI:** 10.1128/mbio.00245-23

**Published:** 2023-03-06

**Authors:** Katrien C. K. Poelaert, Regina G. D. M. van Kleef, Mengying Liu, Arno van Vliet, Heyrhyoung Lyoo, Lora-Sophie Gerber, Yoshiki Narimatsu, Christian Büll, Henrik Clausen, Erik de Vries, Remco H. S. Westerink, Frank J. M. van Kuppeveld

**Affiliations:** a Section of Virology, Division of Infectious Diseases & Immunology, Department of Biomolecular Health Sciences, Faculty of Veterinary Medicine, Utrecht University, Utrecht, The Netherlands; b Neurotoxicology Research Group, Toxicology Division, Institute for Risk Assessment Sciences (IRAS), Faculty of Veterinary Medicine, Utrecht University, Utrecht, The Netherlands; c Copenhagen Center for Glycomics and Department of Cellular and Molecular Medicine and School of Dentistry, Faculty of Health Sciences, University of Copenhagen, Copenhagen, Denmark; Duke University School of Medicine

**Keywords:** EV-D68, neurotropism, receptor

## Abstract

Enterovirus D68 (EV-D68) is an emerging pathogen associated with mild to severe respiratory disease. Since 2014, EV-D68 is also linked to acute flaccid myelitis (AFM), causing paralysis and muscle weakness in children. However, it remains unclear whether this is due to an increased pathogenicity of contemporary EV-D68 clades or increased awareness and detection of this virus. Here, we describe an infection model of primary rat cortical neurons to study the entry, replication, and functional consequences of different EV-D68 strains, including historical and contemporary strains. We demonstrate that sialic acids are important (co)receptors for infection of both neurons and respiratory epithelial cells. Using a collection of glycoengineered isogenic HEK293 cell lines, we show that sialic acids on either N-glycans or glycosphingolipids can be used for infection. Additionally, we show that both excitatory glutamatergic and inhibitory GABA-ergic neurons are susceptible and permissive to historical and contemporary EV-D68 strains. EV-D68 infection of neurons leads to the reorganization of the Golgi-endomembranes forming replication organelles, first in the soma and later in the processes. Finally, we demonstrate that the spontaneous neuronal activity of EV-D68-infected neuronal network cultured on microelectrode arrays (MEA) is decreased, independent of the virus strain. Collectively, our findings provide novel insights into neurotropism and -pathology of different EV-D68 strains, and argue that it is unlikely that increased neurotropism is a recently acquired phenotype of a specific genetic lineage.

## INTRODUCTION

Enteroviruses (EV) are positive-strand RNA viruses within the *Picornaviridae* family and are among the most common human viruses. Poliovirus is the best known and extensively investigated EV. Due to intensive, worldwide vaccination efforts, the incidence of paralytic poliomyelitis has been largely eliminated. EV-D68 is an EV that recently reemerged as an infectious cause of severe respiratory illness in children. Moreover, EV-D68 is associated with a mysterious paralyzing disease, acute flaccid myelitis (AFM), and has therefore been considered a global public health problem since 2014. AFM is a poliomyelitis-like spinal cord syndrome that often results in paralysis and disability in young children. This apparent change in pathogenicity into a neurotropic virus over a relatively short period of time has led to increased interest and awareness of the virus in recent years. Unfortunately, the incidence of EV-D68 worldwide is still underestimated as most infections occur subclinically or cause only mild illness in patients. The EV-D68 genotype can be categorized into four distinct clades (A to D), all of which have diverged from the prototypic strains (i.e., Fermon) isolated in the 1960s, based on the phylogenetic analysis of the VP1 coding region of the genome. Although clade B (diverged from clade C around 2007) and D are often associated with AFM-cases in humans (2014, USA; 2016, the Netherlands) (1), it seems unlikely that neurotropism is a recently acquired phenotype of a specific genetic lineage, as different research groups have reported that both prototypic and contemporary strains from different clades can infect human neurons (2, 3), which are considered the target cells of EV-D68 in the CNS *in vivo* (2).

To date, it is still unidentified how EV-D68 enters and reproduces in the CNS and causes neurological disease. Various studies demonstrated the importance of terminal carbohydrate moieties on the cell surface for EV-D68 infections. A genome-wide genetic screening in human haploid cells revealed that EV-D68 binds to sialic acid receptors, with a preference for α2-6 over α2-3 linked sialic acids (4, 5). Structural studies demonstrated that sialylated glycans bind into the narrow EV-D68 canyon of VP1 on the virus surface, resulting in a series of conformational changes, leading to the displacement of the hydrophobic pocket factor from the VP1 canyon (6). However, this binding did not trigger virus uncoating, suggesting that sialic acids may serve a role as attachment receptor rather than uncoating receptor, and that additional cues are required for efficient virus RNA release and infection (6). Additionally, some EV-D68 strains were identified that could infect cells devoid of sialic acids (4, 5). A genome-wide genetic screen of sialic acid-lacking cells revealed that these strains could use sulfated glycosaminoglycans (GAGs) as attachment receptor, and it was shown that infection of these strains—but not of strains strictly relying on sialic acids—could be blocked by heparin (4, 5). Another study identified the protein intercellular adhesion molecule 5 (ICAM-5), an adhesion molecule expressed on the surface of certain neuronal populations, as an EV-D68 uncoating receptor (7). However, Rosenfeld et al. (2) recently demonstrated that EV-D68 infection did not correlate with the ICAM-5 distribution and expression in human CNS. Moreover, results of that study suggested that EV-D68 neurotropism may be independent of sialic acids. Hence, the identity of the EV-D68 neuronal receptors remains unclear.

Once the viral genome is released into the cytoplasm of the cell, EVs rely on the host cell machinery to replicate their genome and generate viral progeny. Like many (+) RNA viruses, EVs induce drastic rearrangements of the endomembrane structures of the infected cells to form specific intracellular compartments, also called replication organelles (RO), containing viral and host proteins, to facilitate viral RNA replication (8–11). The 3A protein of EVs interact with acyl-CoA binding domain containing 3 (ACBD3) to recruit phosphatidylinositol 4-kinase B (PI4KB) to ROs, resulting in the enrichment of PI4P lipids and, through a PI4P/cholesterol counterflux, ultimately in the enrichment of cholesterol in the RO membranes (10, 11). Until present, it is unknown if similar RO are formed in the soma and/or processes of EV-D68-infected neurons. Moreover, it is largely unknown how these changes in intracellular membranes together with other virus-induced changes in intracellular metabolism—e.g., enteroviral effects on cellular gene expression, cytoskeleton, ion fluxes (5, 8, 12–14)—affect neuronal activity and viability.

In this study, we used primary rat cortical cultures to gain more insight into neurotropism, receptor usage, replication, and the physiological consequences of neuronal infection by historical and contemporary EV-D68 strains.

## RESULTS

### Setup of a primary rat cortical neural infection model.

**FIG 1:**
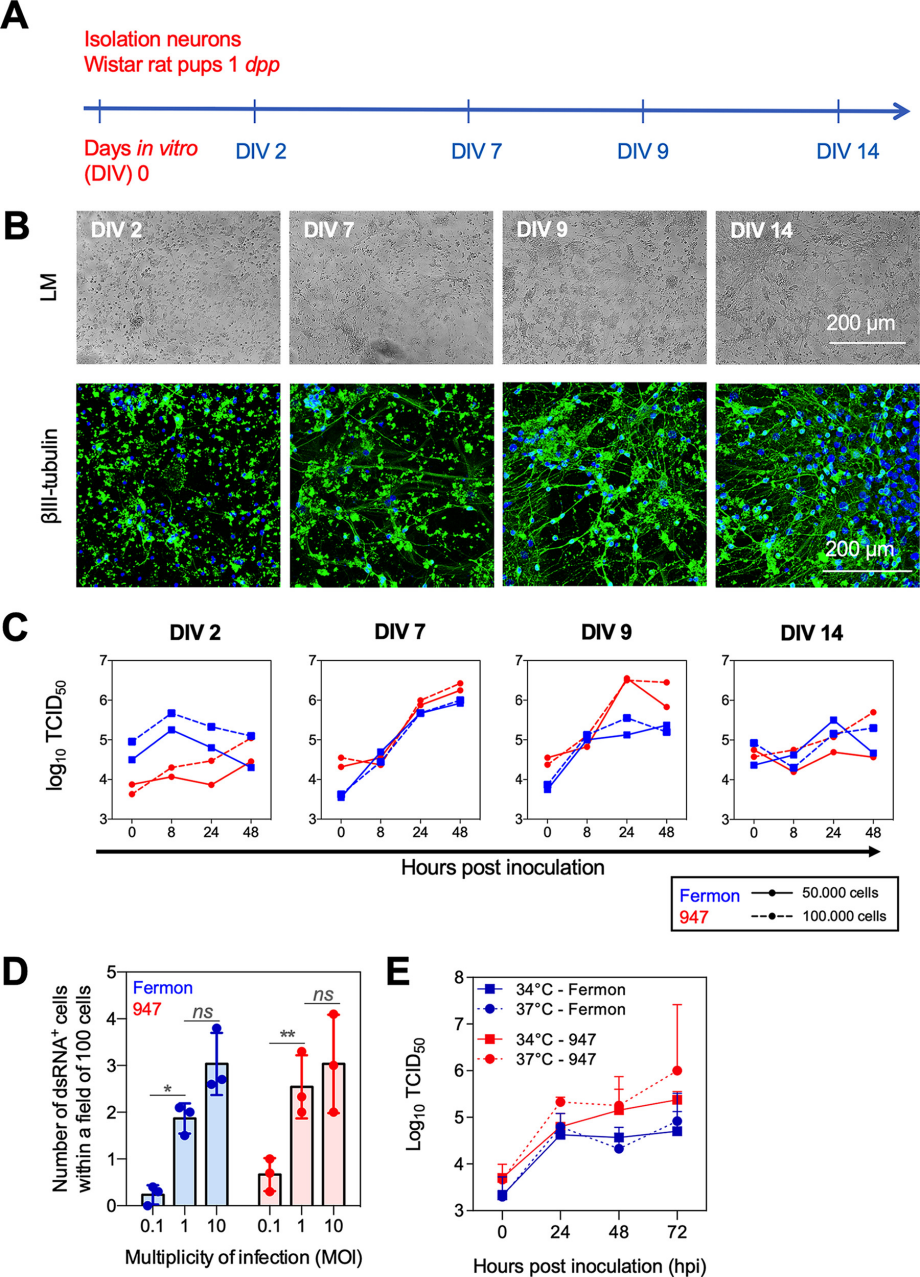
Setup of a primary rat cortical neural infection model. (A) Primary cortical cultures were isolated from Wistar rat pups at 1 day postpartum (dpp). The cells were cultured at a density of 50,000 or 100,000 per well of a 48-well plate, during 2, 7, 9, and 14 days *in vitro* (DIV). (B) Representative light microscopic (LM) images and confocal images of the neuronal network. The neuronal cell marker, βIII tubulin, was stained in green and the nuclei were counter stained in blue. (C) Virus titers of EV-D68-inoculated cortical cultures at days *in vitro* (DIV) 2, 7, 9, and 14, respectively. Blue dots and lines show the results of the prototypic Fermon EV-D68 strain; Red dots and lines show the results of the contemporary 947 EV-D68 strain. (D) Graphical illustration of the number of of dsRNA^+^ cells within a field of 100 cells upon inoculation with Fermon or 947 at a multiplicity of infection (MOI) of 0.1, 1, or 10. 10 fields of 100 cells were analyzed of three independent experiments (E) At DIV 7, the cortical cultures were inoculated and cultivated either at 34 or 37°C. The virus titers of the cell lysates and supernatants combined were quantified over time (hours postinoculation, hpi). Experiments were performed with the supernatants and cell lysates combined of cells derived from three individual cortical isolations. *Ns* indicates not significant; * represents *P* ≤ 0.05; ** represents *P* ≤ 0.01.

To study entry and replication of EV-D68 in neurons and its consequences on cellular activity and viability, an *in vitro* primary rat cortical neuronal infection model was established. Rat cortical cultures were used as they currently represent the gold standard for neuronal activity measurements (15). Fig. 1A illustrates the setup of the neural model. Primary cells were isolated from the cortices of Wistar rat pups at 1 day *postpartum* (dpp), and cultured at a density of 50,000 or 100,000 cells per well of a 48-well plate. Virus replication of one historical strain (i.e., prototypic EV-D68-Fermon) and one circulating contemporary strain (EV-D68-947) was investigated. After 2, 7, 9, and 14 days *in vitro* (DIV), cortical cultures were exposed to the prototypic or contemporary strain to determine the optimal DIV for efficient virus infection (MOI of 1). Cells and supernatants were collected at 0, 8, 24, and 48 h postinfection (hpi) and analyzed by light microscopy, immunofluorescence staining, and virus titrations. Individual rounded cells were observed at DIV 2 (Fig. 1B, left). At DIV 7 and DIV 9, axonal and dendritic processes were formed, resulting in a young neuronal network (Fig. 1B, middle) that becomes more complex and mature at DIV 14 (Fig. 1B, right). The neural density (i.e., 50,000 or 100,000 cells) did not have an effect on the virus titers at 8, 24, or 48 hpi (Fig. 1C). At DIV 2, neither EV-D68-Fermon nor -947 could efficiently infect the cortical cells (Fig. 1C, left). At DIV 7 and DIV 9, both EV-D68 strains could infect and reproduce in the cortical cells, as indicated by a 100-fold increase in virus titer at 48 hpi (Fig. 1C, middle). Infection and reproduction became less efficient at DIV 14, possibly caused by the density of the network (Fig. 1C, right). Subsequently, to assess the optimal MOI of infection, cells were infected with Fermon or 947 at an MOI of 0.1, 1, or 10 at DIV 7. The percentage of infected cells reached a plateau at an MOI of 1 and did not significantly increase at MOI 10 (Fig. 1D). To determine the optimal temperature of incubation, infection assays (DIV 7) were performed at 34 or 37°C. We measured virus titers of whole-cell lysates and supernatants and found no differences between both temperatures (Fig. 1E).

Altogether we demonstrated that at DIV 7 or 9, the neuronal network is most permissive for EV-D68 infection and reproduction *in vitro*. Therefore, in all subsequent experiments, cells were seeded at a density of 100,000 per well and were cultured and infected at an MOI of 1 at DIV 7 at 37°C.

### EV-D68 efficiently reproduces in rat cortical glutamatergic and GABA-ergic neurons.

**FIG 2:**
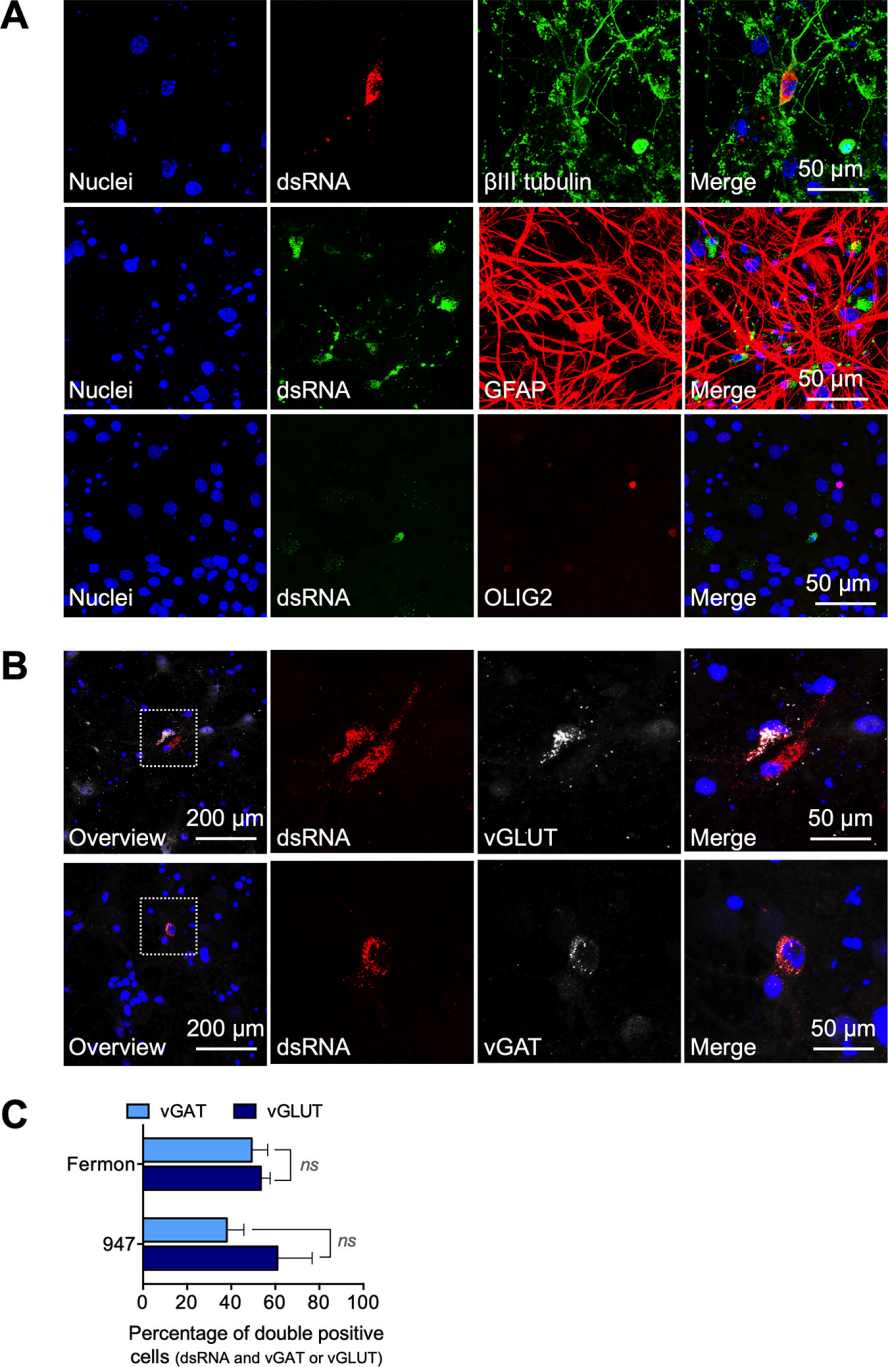
EV-D68 efficiently reproduces in rat cortical glutamatergic and GABA-ergic neurons. Neurons are the predominant infected cell type during EV-D68 infection in the primary cortical cultures *in vitro*. (A) Representative confocal images of the cortical model. The double-stranded RNA (dsRNA) was stained in red and the neural βIII tubulin was stained in green (*upper panel*); dsRNA is shown in green, and the astrocyte-marker Glial fibrillary acidic protein (GFAP, *middle panel*) and oligodendrocyte marker OLIG2 (*lower panel*) are shown in red. In all panels, the nuclei are counterstained in blue. (B) Representative confocal images of the double immunofluorescence staining of the dsRNA marker J2 (red) and the vesicular glutamate transporter (vGLUT, white) or the vesicular GABA transporter (vGAT, white). Nuclei are counterstained in blue. (C) Graphical illustration of the percentage glutamatergic and GABA-ergic neurons, inoculated with Fermon or 947, that express dsRNA. Experiments were performed with cells derived from three individual cortical isolations. *Ns* indicates not significant. Raw data are available in Suppl Table S1.

To demonstrate that neurons become infected with EV-D68, we performed an immunofluorescence staining to visualize viral replication (dsRNA), the specific neuron marker (βIII-tubulin), and the astroglia cell markers (GFAP for astrocytes and OLIG2 for oligodendrocytes). The results (Fig. 2A) confirm that neurons, but not glial cells, become infected. Next, the dsRNA antibody was combined with antibodies specific for excitatory neurons (vesicular glutamate transporter, vGLUT) or inhibitory neurons (vesicular GABA transporter, vGAT) to visualize which neural subpopulation is most susceptible to EV-D68 infection and replication. The results (Fig. 2B and C) demonstrate that Fermon and 947 infect excitatory and inhibitory neurons *in vitro* to a comparable extent.

10.1128/mbio.00245-23.1 TABLE S1 The absolute numbers of dsRNA+, vGAT+ and vGLUT+ cells within a field of 100 cells. Experiments were performed with cells derived from three individual cortical isolations. Download Table S1, TIF file, 0.1 MB. Copyright © 2023 Poelaert et al. 2023 Poelaert et al. https://creativecommons.org/licenses/by/4.0/ This content is distributed under the terms of the Creative Commons Attribution 4.0 International license.

Altogether, we conclude that the prototypic Fermon and contemporary 947 strain efficiently infect and reproduce in glutamatergic and GABA-ergic neurons over time.

### EV-D68 strains differ in their glycan dependency for efficient infection.

Carbohydrate receptors are important (co)receptors for several EVs, including EV-D68 (2, 4, 5, 16–19). Nevertheless, it is currently unknown whether these carbohydrate moieties are EV-D68 (co)receptors in different target cell types, such as neurons and respiratory epithelial cells. Here, primary rat cortical neurons and the human respiratory epithelial Calu-3 cells were either treated with a neuraminidase (NA) to remove sialic acids capping glycans, or with PDMP, which inhibits the glucosylceramide synthase and blocks synthesis of glycolipids, before infection. Additionally, the virus preparation was incubated with glycosaminoglycans (heparin) before infection to determine potential virus binding to heparan sulfate. Coxsackie B3 (CVB3) virus, which does not rely on sialic acids or heparan sulfate for efficient infection, was included as control for the infections of Calu-3 and HEK293 cells. Mouse hepatitis virus (MHV S-Rec), which uses heparan sulfate as a receptor, was included as a control for the infection of rat neurons, as CVB3 failed to efficiently infect these cells.

**FIG 3:**
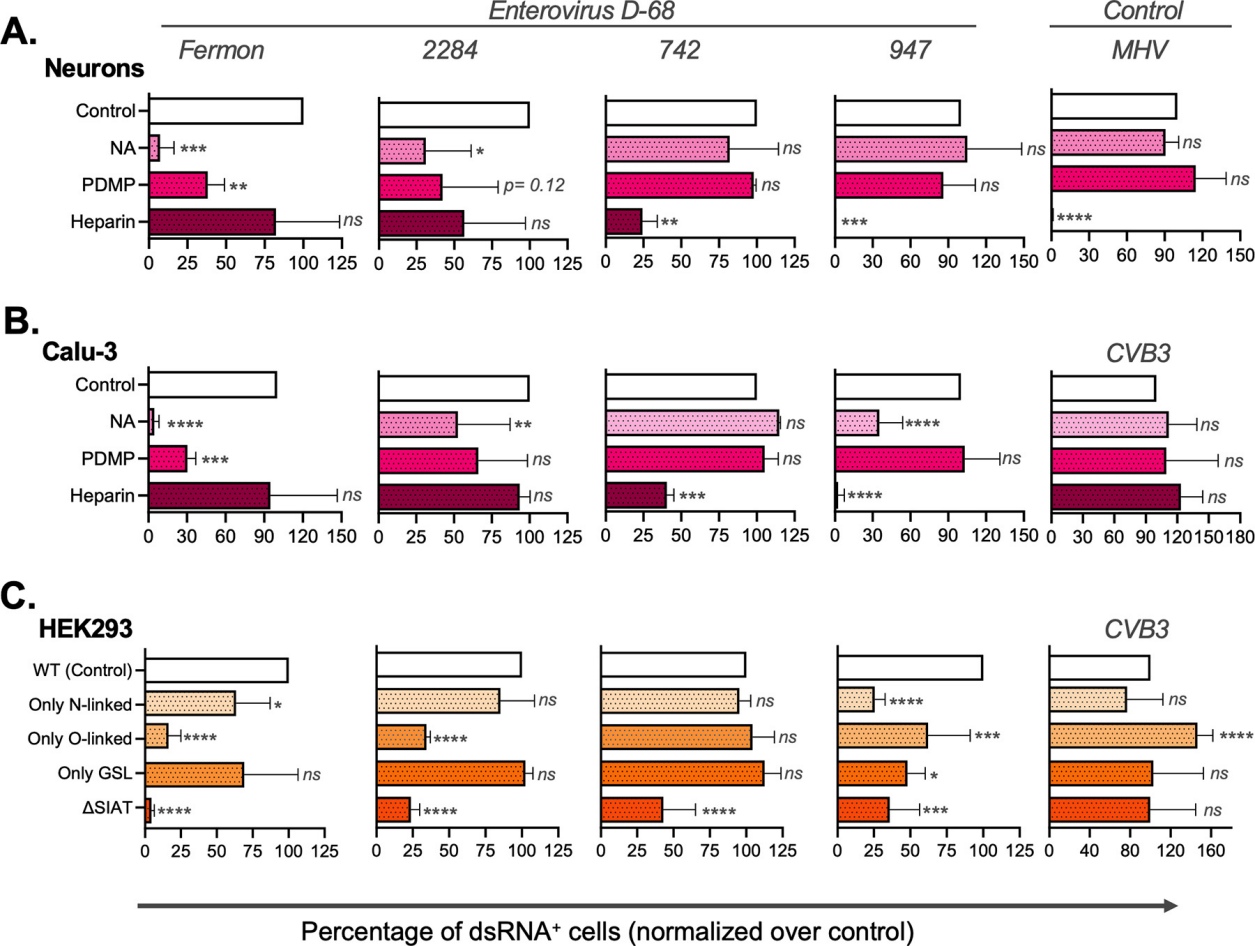
EV-D68 strains differ in their glycan dependency for efficient infection. Cortical cells, Calu-3 cells and HEK293 isogenic cells are included in this figure. Cells were inoculated with Fermon, 2284, 742, or 947 (MOI of 1). The EV-D68 replication in cortical, Calu-3 and HEK293 cells is shown in Suppl Fig. 1. (A) Primary cortical cultures and (B) Calu-3 cells were treated with either neuraminidase or PDMP prior to inoculation. In another condition, the inoculum was treated with heparin. (C) The human isogenic cell lines that lack specific glycosyltransferases were inoculated with Fermon, 2284, 742, or 947 to evaluate the role of certain carbohydrate moieties in EV-D68 entry. *Ns* indicates not significant; * represents *P* ≤ 0.05; ** represents *P* ≤ 0.01; *** represents *P* ≤ 0.001; **** represents *P* ≤ 0.0001.

The prototypic Fermon, and the contemporary 2284, 742, and 947 EV-D68 strains as well as the viral controls (MHV and CVB3) were compared for their ability to infect primary neurons, Calu-3 epithelial cells, and HEK293 cells. The results are shown in Fig. 3 and Fig. S1. Previously, we showed that Fermon and 2284 infect HAP1 cells exclusively in a sialic acid dependent manner, whereas 742 and 947 can also infect HAP1 cells in a heparan sulfate dependent manner in the absence of sialic acids (4). Treatment of neurons with NA of Vibrio cholerae significantly reduced infection with Fermon and 2248 (Fig. 3A, left panels), but not with the 742 and 947 strains (Fig. 3A, right panels). PDMP treatment of neurons strongly reduced infection of Fermon and strain 2284, suggesting that these viruses rely on glycolipids with sialic acid for infection, but not of strains 742 and 947. Notably, pretreatment of the virus with heparin completely blocked the entry of 742 and 947, but not that of Fermon and 2284. The entry of MHV-S-Rec (negative control) in neurons was significantly reduced by pretreatment with heparin, as previously described (20). Comparable results were observed in Calu-3 cells (Fig. 3B), although infection of the 947 strain was reduced upon treatment with NA, hinting to some involvement of sialic acid for efficient entry in these cells. The entry of CVB3 (negative control) was not affected by any enzyme or drug pretreatment (Fig. 3B, right panel). Collectively, these results illustrate that sialic acids are important for EV-D68 infection of neurons and epithelial cells but that some strains (i.e., 742 and 947) can use heparan sulfate for infection, similarly as observed previously in HapI cells (4).

10.1128/mbio.00245-23.2 FIG S1 EV-D68 replication in cortical cells, Calu-3 cells and HEK293 isogenic cells. Number of dsRNA+ cells within a field of 100 cells at 48 hpi. Download FIG S1, TIF file, 0.2 MB. Copyright © 2023 Poelaert et al. 2023 Poelaert et al. https://creativecommons.org/licenses/by/4.0/ This content is distributed under the terms of the Creative Commons Attribution 4.0 International license.

Next, we set out to gain deeper insight into the requirement of the types of glycoconjugates with sialic acids for efficient EV-D68 infection (Fig. 3C). For this, we used human glycoengineered isogenic HEK293 cell lines in which the capacity to produce elaborated glycans (including those with sialic acids) has been eliminated by knockout of glycosyltransferase genes so that they produce only one of the three main types of glycoconjugates (i.e., N-glycans, O-glycans, or glycosphingolipids [GSLs]) with sialic acids. Moreover, we included the HEK293^ΔSIAT^ cell line that elaborates all types of glycans but do not cap with sialic acids. Fermon and strain 2284 could efficiently infect cells expressing only N-glycans or glycosphingolipids, but not cells expressing only O-glycans or cells lacking sialic acids (although strain 2284 was less sensitive to depletion of sialic acids than Fermon). No major differences in infection of cells expressing only N-glycans, O-glycans, or glycosphingolipids by strains 724 and 947 (of which the latter, remarkably, showed less efficient infection of all glycoengineered cell lines) was observed and these viruses were also less sensitive to depletion of sialic acids than the Fermon strain.

Altogether, our data suggest that sialic acids on N-glycans and/or glycosphingolipids are important for infection of HEK293 cells—and possibly also neurons and respiratory cells—by Fermon and 2284, while 742 and 947 are less dependent on sialic acids and can also use heparan sulfate for infection.

### EV-D68 reorganizes intracellular neural membranes in the soma and processes to create a microenvironment for viral replication.

**FIG 4:**
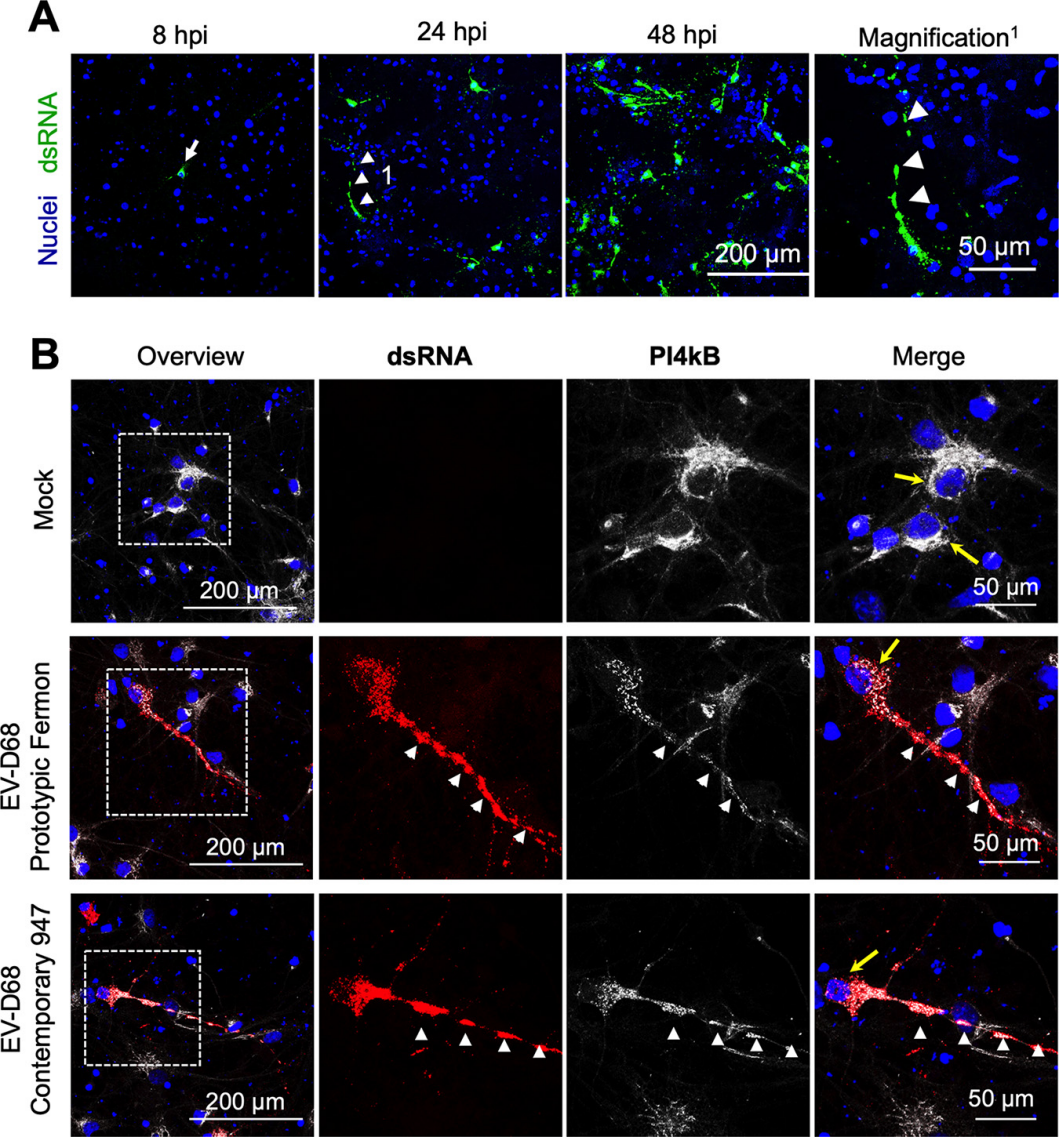
EV-D68 reorganizes intracellular neuronal membranes in the soma and processes. Primary rat cortical cells were inoculated, fixed and cellular or viral markers are immune stained. (A) Representative confocal images of EV-D68 replication at 8, 24 or 48 hpi. The double-stranded RNA (dsRNA) marker J2 is shown in green. The nuclei are counterstained in blue. (B) Representative confocal image: dsRNA J2 marker (red) and the cellular phosphatidylinositol 4-kinase β (PI4kβ, white) and nuclei (blue). Yellow arrows indicate the expression of PI4kβ in the soma of the neurons; white arrowheads show the colocalization of the expression of dsRNA and PI4kβ in the neuronal processes.

Enteroviruses extensively remodel cellular lipid homeostasis and host membranes into specialized membranous organelle-like compartments in the cytoplasm (i.e., replication organelles, RO) where the viral genome is replicated (21). However, whether similar modifications also occur in the soma and/or processes in neurons during EV-D68 replication is yet unknown. To investigate this, neurons were infected with Fermon or 947, fixed at 8, 24 or 48 hpi. First, antibody J2 was used to visualize dsRNA in green. Interestingly, at 8 hpi, EV-D68 replication starts in the soma of the neurons and spreads along the processes around 24 and 48 hpi (Fig. 4A). Then, at 24 hpi infected neurons were fixed and analyzed using IF staining to visualize phosphatidylinositol 4-kinase IIIβ (PI4KB), which is critical in organizing trafficking pathways in eukaryotic cells, including neurons, and is hijacked by EVs in multiple cell types for RO formation (8, 10, 11, 22, 23). In mock-infected neurons, PI4KB is mainly expressed in the soma, and at the most retrograde region of the processes (Fig. 4B). At 24 h postinfection with Fermon and 947, viral replication (dsRNA) was observed in the soma and processes of the neurons. Interestingly, PI4KB relocalized to the viral replication sites, resulting in a clear colocalization of dsRNA and PI4KB in the soma, axons and dendrites (Fig. 4B). No differences were observed between the prototypic Fermon and the contemporary 947 strains.

Overall, these data provide evidence that both prototypic and contemporary EV-D68 strains relocalize PIKB in the soma and recruit this lipid kinase to the processes of neurons, to create a microenvironment for viral replication.

### EV-D68 infection results in a nonapoptotic, necrosis-like cell death in neurons.

**FIG 5:**
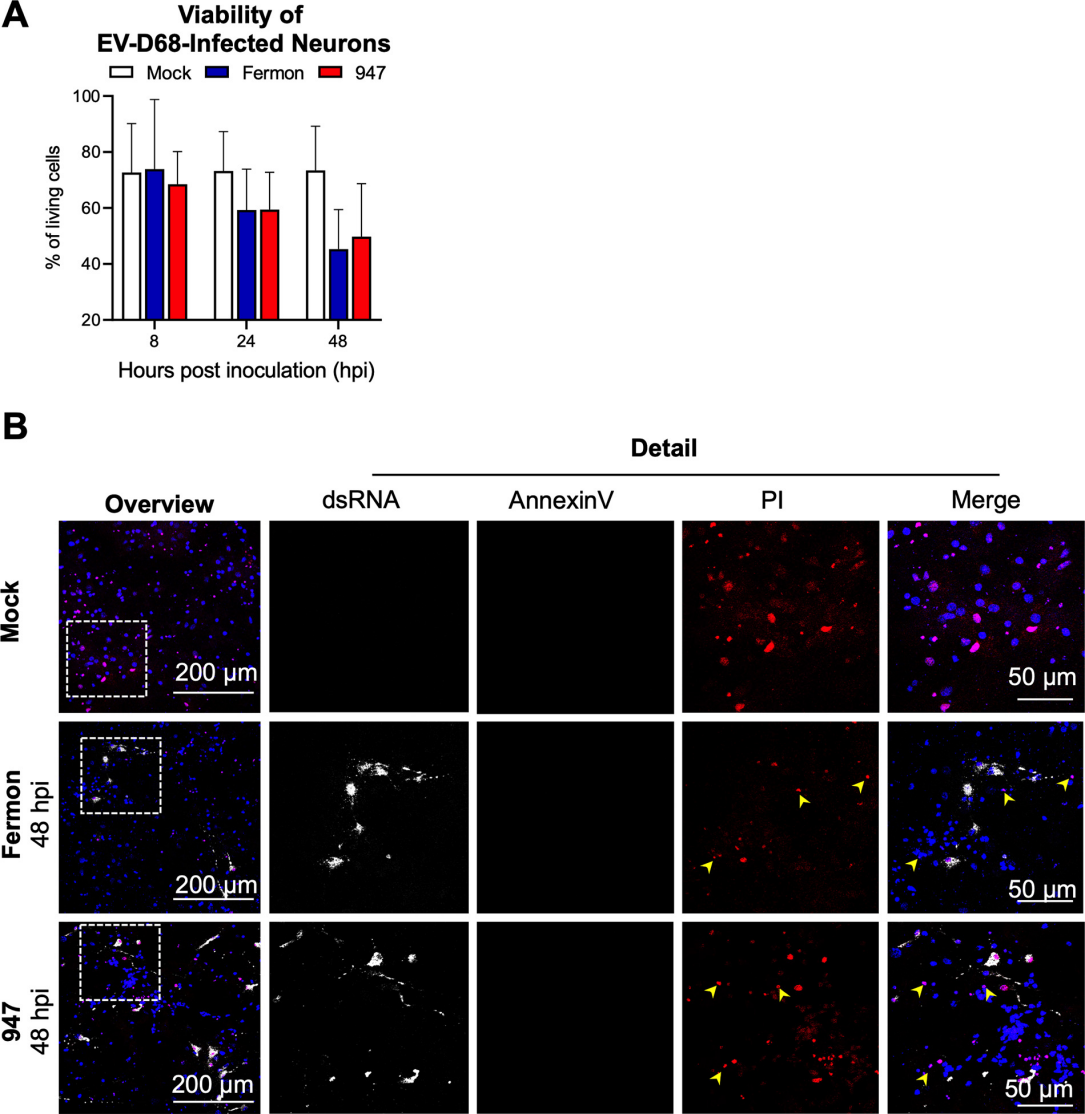
EV-D68 infection results in a nonapoptotic, necrosis-like cell death in neurons. (A) The cell viability of mock-, Fermon- or 947-inoculated neurons over hours postinoculation (hpi). To calculate the cell viability, 10 fields of 100 cells were analyzed of three independent experiments. (B) Representative confocal images at 48 hpi. Annexin V is shown in green, the cell necrosis marker propidium iodide (PI) in red and the dsRNA in white. Yellow arrowheads show the noninfected PI-positive neurons.

The viability of mock- and EV-D68 infected neurons was tested using a cell viability staining kit, in combination with the dsRNA marker J2. Throughout the experiment, about 25% of the mock-infected cells stained positive for cell death. Upon infection, the percentage of nonviable cells increased over time, starting from 24 hpi and reaching a plateau at 48 hpi (Fig. 5A, representative images at 48 hpi are shown in Fig. 5B). To gain more insight into the type of cell death that is induced by EV-D68 infection, we stained cells with AnnexinV (in green), a marker for apoptosis, and propidium iodide (in red), a marker for lytic necrotic cells. The results (Fig. 5B) show that EV-D68 infection of neurons resulted in a nonapoptotic, necrosis-like cell death.

### EV-D68 infection disturbs the spontaneous neuronal activity of the neuronal network *in vitro*.

**FIG 6:**
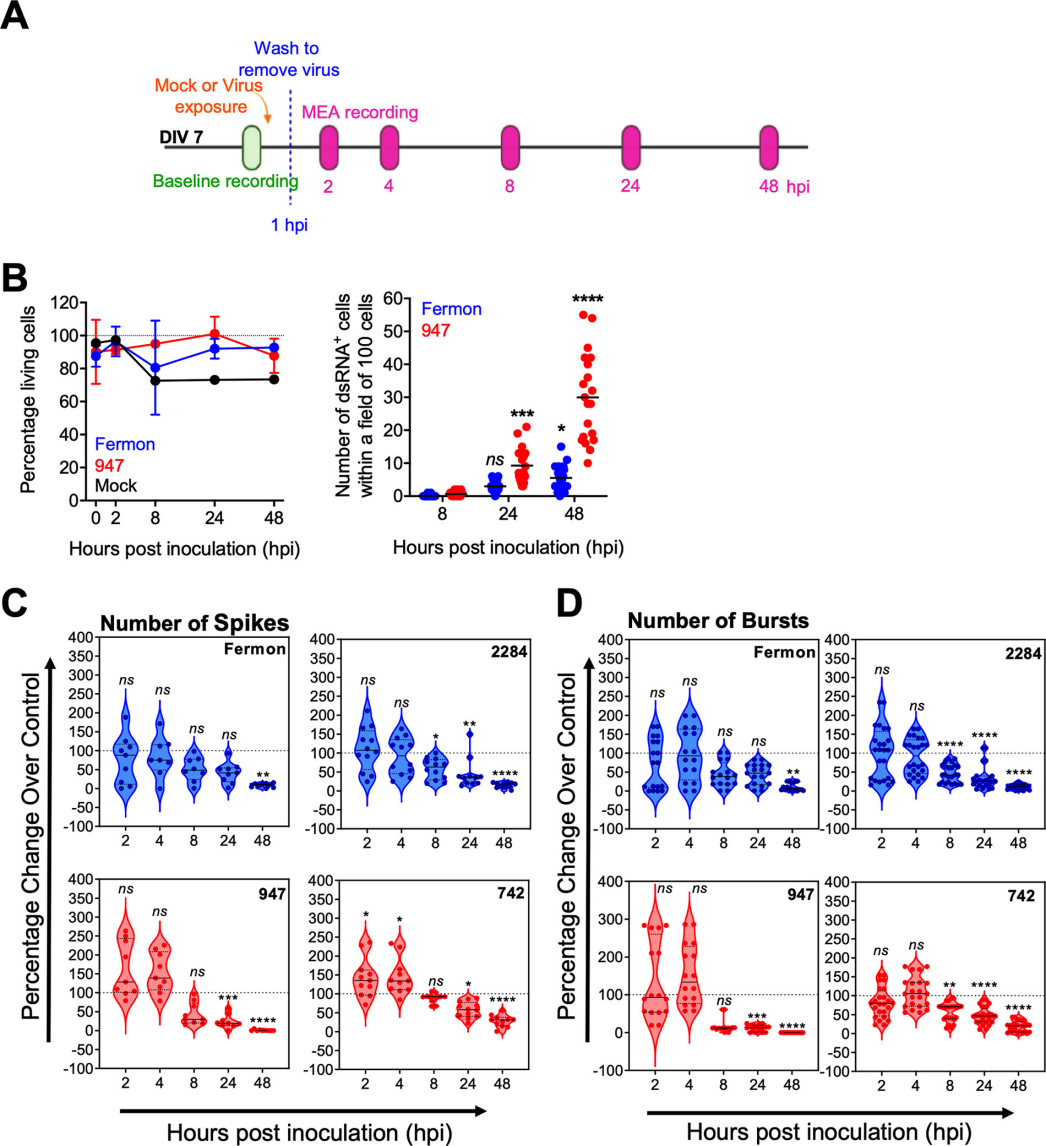
EV-D68 infection disturbs the spontaneous neuronal activity of the neuronal network *in vitro*. Measuring the effects of EV-D68 infection and replication on spontaneous neuronal activity by microelectrode array (MEA) recordings. (A) The experimental setup. Cortical cells were cultured until days *in vitro* (DIV) 7. Prior to mock- or EV-D68-inoculation (MOI of 1, shown in orange), baseline spontaneous neuronal activity was measured (shown in green). At 1 h postinoculation (hpi) the cortical cultures were carefully washed (shown as blue dotted line). At 2, 4, 8, 24, and 48 hpi the spontaneous neuronal activity was measured (shown in pink). (B) The graphical illustration of the percentage of living cells in the neural network of a triplicate of three independent experiments *(left graph)*. The graphical illustration shows the number of neurons expressing dsRNA over time. 10 fields of 100 cells spread over three independent experiments were analyzed *(right graph)*. (C and D) The graphical illustration of the MEA recordings of 16 wells of each condition. (C) The number of spikes is shown as percentage change relative to the control in time postinoculation (hpi). (D) The number of bursts is shown as percentage change relative to the control in time postinoculation (hpi). All data shown in blue are EV-D68 strains that use a carbohydrate receptor for viral entry; All data shown in red are EV-D68 strains that mainly bind to heparan sulfate for viral entry. *Ns* indicates not significant; * represents *P* ≤ 0.05; ** represents *P* ≤ 0.01; *** represents *P* ≤ 0.001; **** represents *P* ≤ 0.0001.

We next analyzed the physiological consequences of EV-D68 infection and replication on the spontaneous neuronal activity. For this, we used microelectrode array (MEA) recordings. Fig. 6A illustrates the experimental set up. The spontaneous neuronal baseline activity was recorded prior to the exposure to the virus. Then, cells were infected with EV-D68 and incubated during 1h at 37°C. Next, cells were carefully washed to remove nonadherent virus particles, and at different time points postinfection, the spontaneous neuronal activity was measured.

Fig. 6B shows the viability of the whole cortical network (neurons and astroglia cells) upon mock- or EV-D68 infection (left panel) and the number of infected neurons over time (right panel). Although 80% of the whole cortical network remains viable, a large increase in the number of infected neurons was observed over time for both Fermon and 947. In Fig. 6C and D, the EV-D68-infected cortical neurons were grown in wells of a MEA plate, and the spontaneous neuronal activity pattern was analyzed over time postinfection. In these sets of experiments, we included the historical Fermon and the contemporary 2284 strain, both of which rely on sialic acid, as well as the contemporary strains 947 and 742 that can also use heparan sulfate to infect neurons. The activity pattern includes spiking (Fig. 6C) and bursting (Fig. 6D). Spikes represent the extracellular field recordings of action potentials. At early time points (i.e., at 2 and 4 hpi), no large alterations in the number of spikes were seen for all EV-D68 strains, whereas at later time points (i.e., from 8 hpi onwards), a reduced firing rate of action potentials was observed. Bursting is a sequence of spikes with a short interval measured by one single electrode. In line with the effects observed for spiking, the number of bursts decreased in the networks infected with all EV-D68 strains (Fig. 6D) from 8 hpi onwards. Altogether, we conclude that all EV-D68 strains, independent of the clade, origin (prototypic versus contemporary), or receptor reduce the spontaneous neuronal activity.

## DISCUSSION

During the global expansion of EV-D68 in the early 2000s, EV-D68 respiratory disease was associated with the emergence of acute flaccid myelitis (AFM) predominantly in children. Phylodynamic analyses of VP1 sequences of EV-D68 isolates revealed an antigenic drift of the modern circulating viral strains compared to the historic Fermon strain that was isolated more than 50 years ago, implicating an increased diversity of EV-D68 (1, 24). These newly circulating EV-D68 strains have a deletion in the 5’UTR between the IRES and the start of the polyprotein and are diverged into three clades (A, B, and C) due to other small variations in their genomic RNA. To gain more insight into EV-D68 neuropathology, we studied neurotropism, receptor usage, replication, viral effects on neuronal activity, and cell death upon infection of primary cortical cultures by the historic Fermon strain and three circulating strains, namely, 742 (Clade A), 947 (Clade B), and 2284 (Clade C).

Here, we show that these strains use sialic acid on N-glycans and glycosphingolipids as receptor to infect cells. Moreover, we demonstrate for the first time that EV-D68 rearranges the intracellular neuronal membranes to form replication organelles in the soma and later in the processes for viral reproduction. Interestingly, both excitatory and inhibitory neurons are susceptible to EV-D68 infection and support viral reproduction, which results in reduced spontaneous neuronal activity and ultimately causes nonapoptotic, necrosis-like cell death. No major differences were observed among the different EV-D68 strains, arguing against the possibility that the neuropathology is a recently acquired trait that is linked to a specific clade.

Various neuronal models can be used to study viral neurotropism. For example, human-induced pluripotent stem cells (hiPSC) can be exposed to growth factors to differentiate into human neurons and glial cells (25). Yet they are expensive, time-consuming, and not well-characterized. Indeed, early papers suggest that neurons derived from iPSC form immature synapses, only a fraction of the cells fire action potentials, and the cells express markers of immature neurons. Therefore, rat primary cortical cultures are a reliable alternative to use as a model. This rodent model represents the *in vivo* cellular diversity, consisting of excitatory and inhibitory neurons and supportive glial cells (26, 27). Moreover, they have been developed *in situ*, leading to a more mature and reliable functional neuronal network, and are considered the gold standard for neuro-activity testing (28). Based on minor differences between rat and human-derived cells, the low costs, and short culture duration, rat cortical neurons were used to study EV-D68 neuropathogenesis. In this rat cortical neuronal model, we demonstrated that neurons, but not glial cells, become infected with historical and circulating EV-D68. Moreover, in contrast to previous studies, we demonstrated that both excitatory and inhibitory neurons can be infected by both prototypic and contemporary EV-D68 strains. As mentioned above, different research groups reported a phenotypic shift from nonneurotropic to neurotropic viruses in 2014. These studies were conducted *in vivo* in mice, in the neuroblastoma-derived neuronal cell line (SH-SY5Y), as well as human postnatal cortical neuron cultures (3, 16). On the other hand, the study of Rosenfeld et al. (2) demonstrated that, in organotypic mouse brain slices cultures and iPS derived cortical neurons, neurons and astrocytes become infected by both prototypic and contemporary EV-D68 strains. This is partially in line with the observations in our neuronal model, as we also observed that both prototypic and contemporary EV-D68 strains can infect rat cortical neurons, although we did not find any differences in the neurotropism of prototypic or contemporary strains during cultivation at 34 or 37°C. It is important to note that a limitation of our study was the relatively low number of infected primary cells. Moreover, we encountered quite some variation between biological replicates (i.e., rat cortical neurons isolated from different litters), impeding a direct comparison of the absolute number of infected cells in different experiments (e.g., Fig. 1 and Fig. 6). We observed no infection of astrocytes *in vitro.* All in all, it can be concluded that different models yield different, yet possibly important outcomes, which are not easy to reconcile to the situation in humans.

One of the key steps in understanding EV-D68 neurotropism is identifying cellular (co)receptors for viral entry in neurons. Consistent with the haploid screen of Baggen et al. (1), we demonstrated the importance of sialic acid for EV-D68 infection of neural *in vitro*. A similar dependence on sialic acid was observed on human lung epithelial Calu-3 cells as well as on HEK293 cells. Using a collection of glycoengineered HEK293 isogenic cell lines elaborating only N-glycans (HEK293^ΔCOSMC/B4GALT5/6^), only O-glycans (HEK293^ΔMGAT1/B4GALT5/6^), or only glycosphingolipids (GSLs) (HEK293^ΔMGAT1/COSMC^), we demonstrated select differences for sialic acid on N- and O-glycoproteins and GSLs for entry of the tested EV-D68 strains. Efficient infection of Fermon and strain 2284 could be mediated by either N-glycans or GSLs, pointing to redundancy. GSLs are known as receptors for cellular molecules (hormones and interferons), toxins, bacteria, and nonenveloped viruses (caliciviruses, parvoviruses, and reoviruses) (29).

Our study is the first to demonstrate the importance of GSLs in EV-D68 infection. Similar as observed on HapI cells (1), strains 742 and 947 could infect neurons as well as Calu-3 and HEK293 cells in the absence of sialic acids, most likely by using heparan sulfate as receptor. Altogether, these data reveal that important new insights into the glycan receptors that can be used by EV-D68 to infect neural cells.

Previous studies demonstrated that enteroviruses modify host-cell endomembranes to form novel structures that serve as platforms, also called replication organelles (RO), for viral replication (14, 30–35). Specific EV proteins recruit host factors, such as phosphatidylinositol 4-kinase type IIIβ (PI4KB), to the RO (32, 35). However, whether this is also the case for EV-D68 infection in neurons was until present unknown. In this study, neurons were infected with EV-D68, and the kinetics of the EV-D68 replication was checked over time. Interestingly, the EV-D68 reproduction of historical and circulating strains starts in the soma of the infected neurons at 8 hpi. At this moment, PI4kB relocates from the Golgi apparatus into the soma of the infected neuron and colocalizes with the viral replication site. At 24 hpi, viral reproduction and PI4kB relocate to the processes of the infected neurons. This suggests that the endomembranes of the Golgi apparatus and the Golgi outpost in the soma and processes, respectively, are reorganized. The Golgi outpost is a satellite organelle specialized to organize the architecture of the neuronal processes (36). Their exact role in EV-D68 replication in neurons and the effects of their reorganization on neural activity is currently unknown.

Having established that EV-D68 efficiently infects and replicates in rat cortical neurons, we next focused on the spontaneous activity of the neuronal network upon EV-D68 infection. For this, we used microelectrode array (MEA) recordings, a robust, noninvasive, and cutting-edge technology that is often used in neurotoxicology (37). Using this innovative technology, we measured the number of spikes and bursts at 2, 4, 8, 24, and 48 h after infection with the historical (Fermon) and three circulating (2284, 947, and 742) strains. We did not observe any change in neural activity compared to the baseline at 2 and 4 hpi, suggesting that the receptor binding and viral entry do not affect spontaneous neuronal network activity. Around 8 hpi, the number of spikes and bursts of the neuronal network decreased to half of their physiological baseline, independent of the viral strain, which is much earlier than the first signs of nonapoptotic necrosis-like cell death (24 hpi). These data suggest that the neuronal activity is disturbed by viral replication or virus-induced structural reorganization of the intracellular membranes. Importantly, we did not observe any differences in the effects imposed by the historical and the contemporary circulating strains. These data imply that the association of EV-D68 with neurological disease is unlikely due to specific changes in virus strains to infect neuronal cells, but more likely due to increased ability of the virus to reach the CNS, or due to increased circulation of EV-D68 in the population.

## MATERIALS AND METHODS

### Chemicals and reagents.

The following chemicals and reagents were used in this study: Vibrio cholerae neuraminidase (Roche, 11080725001); DL-threo-PDMP (Sigma-Aldrich, 513100); Heparin (Sigma-Aldrich, H4784).

### Viruses.

EV-D68-Fermon (CA62-1; Prototypic strain, EV-D68-742 (4311000742; Clade A), EV-D68-947 (4310900947; Clade B) and EV-D68-2284 (4310902284; Clade C)) were described previously (4, 5) and were obtained from the National Institute of Public Health and the Environment. CVB3 (Nancy strain) was obtained by transfecting *in vitro*-transcribed RNA derived from full-length infectious clone p53CB3/T7 (38). Murine hepatitis virus (MHV)-S-Rec-eGFP was obtained by targeted recombination of the S gene sequence, which uses heparan sulfate as entry receptor (20). For all viruses, low-passage-number stocks were used to avoid cell culture adaptations.

### Primary rat cortical cultures.

Animal experiments were performed in agreement with the Dutch law, the European Community directives regulating animal research (2010/63/EU) and approved by the Ethical Committee for Animal Experiments of Utrecht University. One-day old pups from Wistar rats (Envigo, Horst, The Netherlands) were sacrificed and cortical cultures consisting of neurons and glial cells astrocytes were prepared as described previously (39). First, cells were cultured in dissection medium containing Neurobasal, a medium (NB-A, Gibco, Paisley, UK), 2.8% sucrose (Sigma-Aldrich, S8501, Darmstadt, Germany), 200 mM l-glutamine (Gibco, 25030-024), 3.5 mM glutamate (Sigma-Aldrich), 1% penicillin/streptomycin (P/S), and 1× B-27 supplement (Life Technologies), to maintain neuronal differentiation (pH of 7.4). At day *in vitro* (DIV) 4, the glutamate-containing dissection medium was replaced with glutamate-free culturing medium containing all components of the dissection medium, except glutamate. In all experiments to setup the neuronal model, cortical cells were seeded at a density of 50,000 or 100,000 cells per well in a 48-well plate (Corning Life Sciences, Lasne, Belgium), as indicated. For immunofluorescence (IF) staining, coverslips (12 mm diameter, Gerhard Menzel GmbH, Braunschweig, Germany) were coated with 0.1% polyethyleneimine (PEI; Fluka, P3143) solution diluted in borate buffer (24 mM sodium borate/50 mM boric acid, pH 8.4) during 1h at room temperature (RT). Next, coverslips were washed four times with sterile laboratory-grade water (B. Braun, Melsungen AG, Melsungen, Germany) prior to adding 200,000 cortical cells per well of a 24-well plate. For MEA recordings, 48-well MEA plates (Axion BioSystems Inc., Atlanta, USA) were coated with 0.1% PEI, and cortical cultures were seeded at a density of 100,000 cells per well in a drop as described previously (40). For the cell viability assay of the complete network, cells were cultured at a concentration of 20,000 cells per well of a 96-well plate, coated with PEI. For PEI coating, 0.1% PEI solution was added to the plates/coverslips and incubated for 1 h at room temperature, followed by aspiration of the PEI solution, rinsing 4 times with sterile laboratory-grade water, and air-drying the plates/coverslips overnight at room temperature.

### Cell lines.

Glycoengineered isogenic HEK293 cell lines were generated and characterized as previously described as part of a cell-based glycan array resource (41–43). We employed glycoengineered HEK293 cells that express sialic acids (both α2-3 and α2-6) only on glycolipids (HEK293^ΔMGAT1/COSMC^), only on N-glycans (HEK293^ΔCOSMC/B4GALT5/6^), or only on O-glycans (HEK293^ΔMGAT1/B4GALT5/6^), because the key glycosyltransferase genes required for elongation of these glycosylation pathways were knocked out. We further used the HEK293^ΔSIAT^ cell with knockout of all sialyltransferase genes involved in α2-3 and α2-6 sialylation of glycolipids and glycoproteins (KO of ST3GAL1-6, ST6GAL1/2 and ST6GALNAC1-6), which is essentially devoid sialic acids on these types of glycoconjugates (43). HEK293 cells were cultured in Dulbecco’s Modified Eagle Medium (DMEM, Lonza) supplemented with 10% (vol/vol) FCS and 5% nonessential amino acids (Gibco) (19). All cell lines were tested for mycoplasma contamination. Calu-3 cells (ATCC HTB-55TM, VA, USA) were cultured in DMEM supplemented with 20% (vol/vol) FCS. Rhabdomyosarcoma (RD) cells were obtained from the European Collection of Cell Cultures (cat. No. 85111502) and cultured in DMEM supplemented with 10% (vol/vol) FCS. RD cells were used to grow EV-D68 virus stocks and for virus titration.

**(i) Infectivity assays.** Primary rat cortical cells, Calu-3 cells and all HEK293 isogenic cells were infected with EV-D68 at a multiplicity of infection (MOI) of 0.1, 1, or 10, for 1 h at 34 or 37°C, as indicated. In parallel, cells were infected with MHV-S-Rec-eGFP or CVB3 as a control, at an MOI of 1 for 1 h, at 37°C. After 1 h, the virus was removed, and cells were rinsed three times with culture medium. Fresh culture medium was added and at indicated time points, 0, 8, 24, or 48 hpi, cells were fixed with 4% paraformaldehyde; in the virus production assays, cells were scraped from the plate using a 200 μL pipette tip, supernatants and cells were collected in a 1.5 mL Eppendorf tube, and all tubes were subjected to three freeze/thaw cycles and virus titers were determined by endpoint dilution. In the entry assay, all cell types were treated with 20 μM DL-threo-PDMP during 36 h, or with 100 mM Neuraminidase during 1 h prior to virus infection.

**(ii) Cell viability assays.** Viability of EV-D68-infected cortical cells. The Annexin V-FITC Apoptosis Detection kit (Sigma-Aldrich, APOAF) was used in combination with the dsRNA marker J2 (MAb, mouse, IgG2a, kappa chain; English & Scientific Consulting Kft., Hungary) to detect infected apoptotic cells. The Annexin V-FITC binds to phosphatidylserine in the membrane of apoptotic cells, and propidium iodide (PI) binds to the cellular DNA in cells where the cell membranes have been totally compromised. At the indicated time points postinfection, medium was removed from the cells, the cells were rinsed once with PBS and 500 μL 1× Binding buffer was added to the cells. Next, 5 μL of Annexin V FITC and 10 μL PI solution was added to each well, incubated during 10 min, RT protected from the light. Subsequently, the cells were washed using PBS, and fixed using 4% paraformaldehyde (PFA) diluted in 1× Binding Buffer, 15 min, RT. Cells were permeabilized using 0.01% Triton-X diluted in 1× Binding Buffer, 10 min, RT and washed with PBS prior to the incubation with the dsRNA marker J2, during 1 h at RT on a rocking platform. The Alexa Fluor 647-labeled goat anti-mouse IgG antibody was used to visualize the dsRNA, 1 h, RT. Nuclei were stained with DAPI.

Viability of the primary rat cortical culture. The CellTiter 96 Aqueous One Solution Cell Proliferation Assay (Promega Corporation, G3582, WI, USA) was used as a colorimetric method for determining the number of viable cells in each well of a 96-well plate and was carried out as described by the manufacturer’s protocol. Briefly, 20 μL of CellTiter 96 AQeous One Solution Reagent was added to each well of the 96-well plate containing 100 μL culture medium. The plate was incubated during 3 h at 37°C and 5% CO2 and the absorbance was measured using a 96-well plate reader (BioTek Instruments, Inc., VC, USA).

### Microelectrode array (MEA) recordings and analyses.

The neuronal activity of EV-D68 infected primary rat cortical cultures was analyzed using MEA recordings as described previously (40, 44). First, spontaneous baseline neuronal activity was measured for 30 min (min). EV-D68-Fermon, -947, or culture medium (mock) were added to the culture during 1 h at 37°C, and then cells were washed to remove unbound virus particles. After another hour of incubation (2 hpi), spontaneous neuronal network activity was measured again for 35 min. This measurement was repeated at 4, 8, 24, and 48 hpi. To ensure the reliability of the data, 9-12 wells derived from 2 independent cultures were included for each condition. The analysis of the MEA data were carried out as described previously (18, 22). For each time point, the parameters describing neuronal (network) activity during EV-D68 or mock infection were analyzed during the last 10 min of each MEA recording and expressed as the ratio of exposure (numerator) over baseline (denominator) as a percentage of mock-infected control wells. The outlier analysis was performed as described by Gerber et al. (40). The ratios of exposure were averaged per parameter (number of spikes and number of bursts), per condition (EV-D68 strains), and time point post exposure (2, 4, 8, 24, or 48 hpi). The spontaneous neuronal activity is shown as percentage over control and expressed as mean ± SEM of n_wells_.

### Immunofluorescence assays.

Cell cultures were rinsed three times in PBS and treated with 10% normal goat serum (Gibco) and 10% FBS diluted in PBS (i.e., blocking buffer, 45 min, RT). Viral replication was detected using the monoclonal mouse anti-double-stranded RNA (dsRNA) (J2) antibody (1/1000; Exalpha Biologicals INC., MA, USA) or rabbit anti-dsRNA (J2) antibody (1/25; Absolute Antibody Ltd., UK) diluted in blocking buffer (60 min, RT), followed by incubation with directly conjugated Alexa Fluor 594 secondary antibody (1/200; Life Technologies). Viral capsid proteins were stained using rabbit anti-capsid serum against EV-D68-Fermon (1/1000; produced in-house) or EV-D68-947 (1/100; produced in-house), followed by incubation with the directly conjugated Alexa Fluor 594 secondary antibody (1/200). Neurons, astrocytes or oligodendrocytes were labeled with polyclonal rabbit βIII Tubulin (1/1000; ab18207; Abcam), polyclonal rabbit Glial fibrillary acidic protein (GFAP; 1/2500; NB300-141; Novus Biologicals, Abington, UK), or polyclonal rabbit Olig2 (OLIG2; 1/100; ab9610, Millipore, Amsterdam, NL) antibodies, respectively, followed by incubation with directly conjugated Alexa Fluor 488 or 594 secondary antibodies (1/200; Life Technologies). PI4-Kinase β (PI4kB) was labeled with polyclonal rabbit PI4kB (1/100; 06-578, Merck KGaA, Darmstadt, Germany) followed by incubation with directly conjugated Alexa Fluor 647. Nuclei were counterstained with DAPI (1/1000; Thermofisher) diluted in PBS. All cell cultures were mounted in ProLong Diamond Antifade Mountant (P36970, Invitrogen) and sealed. Images were captured at a magnification of ×20 or ×40 using an inverted Leica SPE-III confocal microscope.

### Statistical analysis.

Data representation and statistical analysis were performed using GraphPad Prism 9 software. Statistical analysis was performed by two-way analysis of variance (ANOVA) followed by a multiple-comparison test unless otherwise specified. A value of *P* < 0.05 was considered statistically significant.
