# Supplementary figures and images for: Enterovirus D-68 Infection of Primary Rat Cortical Neurons: Entry, Replication, and Functional Consequences
Source: mBio. 2023 Mar 6;14(2):e00245-23. doi: 10.1128/mbio.00245-23 (PMC10127580; doi:10.1128/mbio.00245-23)

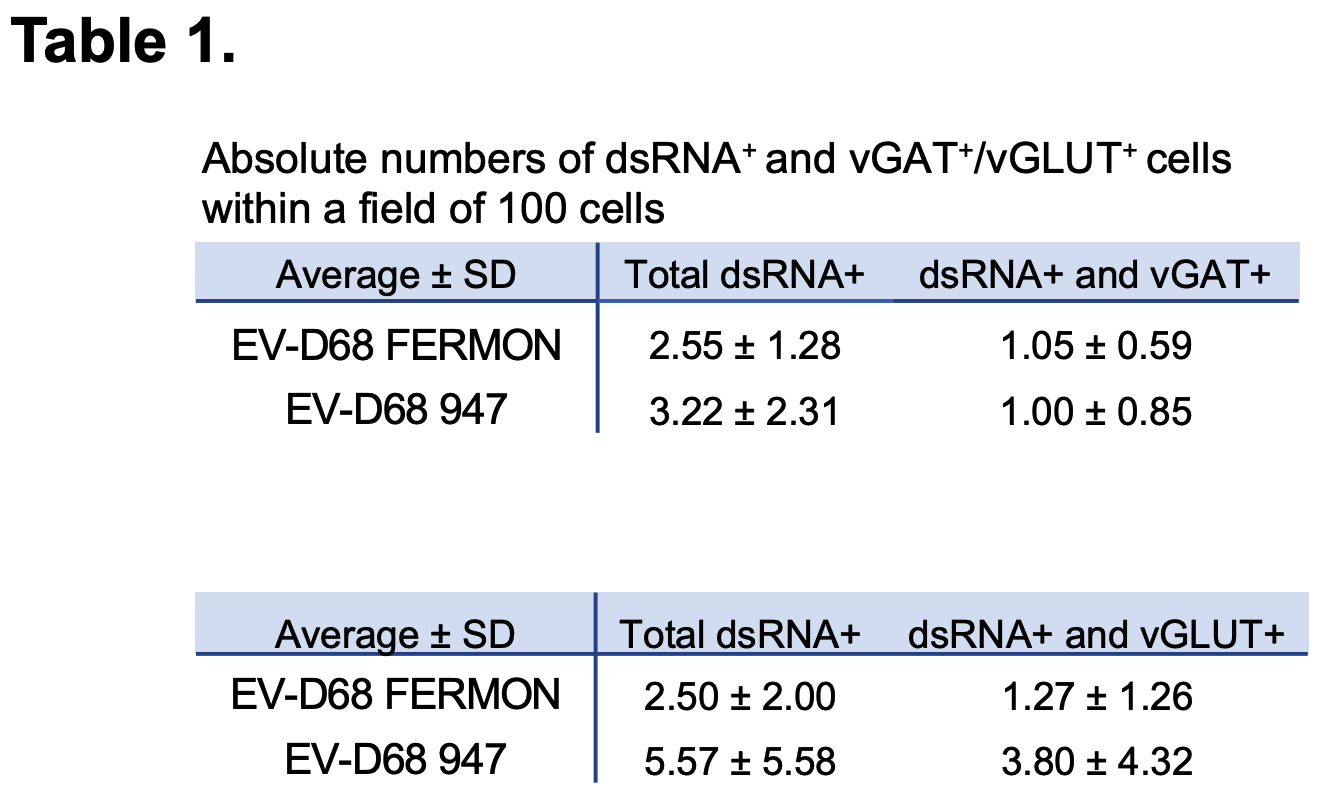

Supplement: TABLE S1 [file mbio.00245-23-s0002.tif]

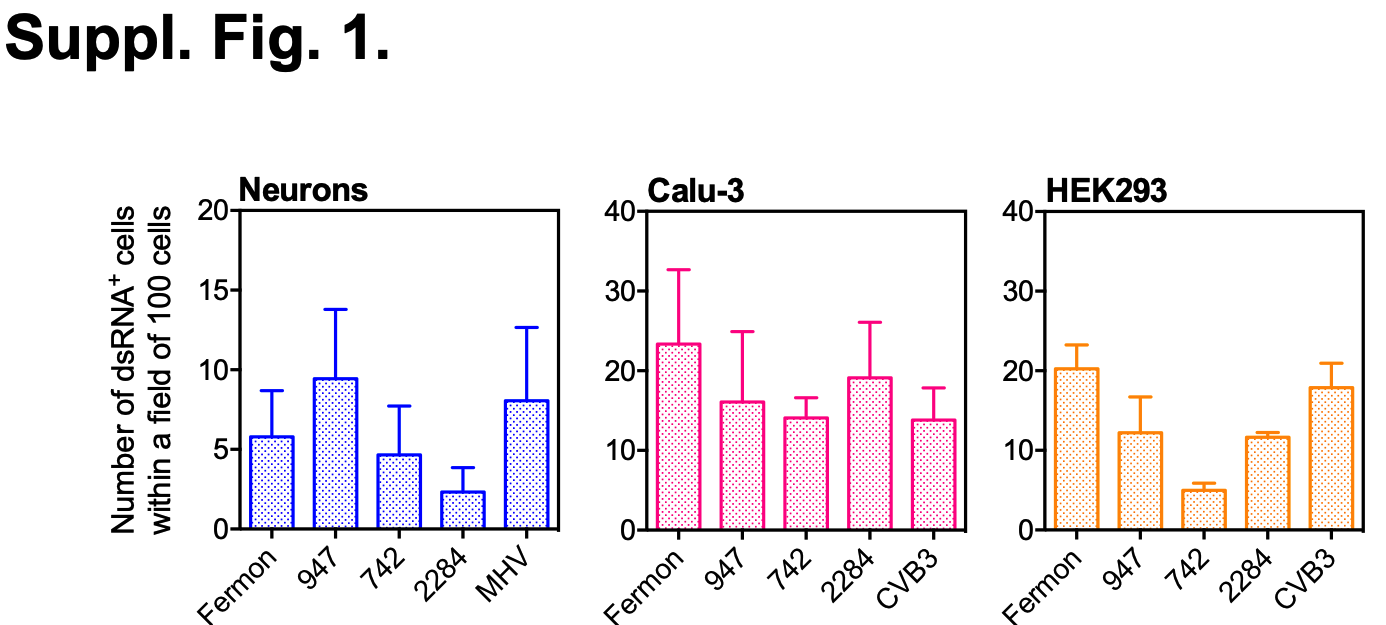

Supplement: FIG S1 [file mbio.00245-23-s0001.tif]
